# Prolyl hydroxylase 2 (PHD2) inhibition protects human renal epithelial cells and mice kidney from hypoxia injury

**DOI:** 10.18632/oncotarget.11104

**Published:** 2016-08-05

**Authors:** Yi Fang, Hui Zhang, Yihong Zhong, Xiaoqiang Ding

**Affiliations:** ^1^ Department of Nephrology, Zhongshan Hospital, Fudan University, Shanghai, China; ^2^ Shanghai Institute of Kidney and Dialysis, Shanghai, China; ^3^ Shanghai Key Laboratory of Kidney and Blood Purification, Shanghai, China; ^4^ Department of Nephrology, The Affiliated Hospital of Qingdao University, Qingdao, China

**Keywords:** prolyl hydroxylase 2, hypoxia inducible factor, renal ischemia-reperfusion injury, autophagy and apoptosis, Pathology Section

## Abstract

Prolyl hydroxylase domain protein 2 (PHD2) is a key oxygen sensor, setting low steady-state level of hypoxia-inducible factor-α (HIF-α). Here, we showed that treatment of cobalt chloride (CoCl_2_), a hypoxia mimic, in HK-2 tubular epithelial cells induced PHD2 and HIF-1/2α expression as well as cell apoptosis and autophagy activation. Three methyladenine (3-MA), the autophagy inhibitor, blocked autophagy and protected HK-2 cells from CoCl_2_. Significantly, siRNA knockdown of PHD2 also protected HK-2 cells from CoCl_2_, possibly via increasing HIF-1α expression. Reversely, HIF-1α siRNA knockdown almost abolished cytoprotection by PHD2 siRNA in CoCl_2_-treated HK-2 cells. *In vivo*, pretreatment with a PHD inhibitor L-mimosine remarkably attenuated mice renal ischemia-reperfusion injuries. Molecularly, L-mimosine inhibited apoptosis and inflammatory responses in injured mice kidneys. Together, our results suggest that PHD2 silence or inhibition protects human renal epithelial cells and mice kidney from hypoxia injuries.

## INTRODUCTION

Acute kidney injury (AKI) is a deterioration of renal function over a period of hours to days [1, 2]. It will cause severe morbidity if not handled properly [1, 2]. Ischemic-hypoxia injury is the major pathological mechanism of AKI [1–4]. Hypoxia-inducible factor-1α (HIF-1α) is a key regulator in adaptation to low oxygen availability [3–5]. It promotes the transcription of many genes involved in cellular and systemic responses to hypoxia [5, 6]. Under normoxia, HIF-1α is degraded by the prolyl-4-hydroxylase domain (PHD) family proteins, which require di-oxygen and 2-oxoglutarate as co-substrates [7].

Thus far three different PHDs have been identified, including PHD1, PHD2, and PHD3 [4, 8]. PHDs have different but sometime overlapping functions. PHD2 is the critical oxygen sensor of HIF-1α in normoxia and hypoxia [8, 9]. The characteristics of theses enzymes provide potential targets for small molecule manipulation, which could activate HIF in the presence of normoxia. For example, cobalt chloride (CoCl_2_) and other iron chelators have been applied to activate HIFs [10, 11]. Existing evidences have shown that enhancing HIF via PHD inhibition could protect against ischemic injuries [11, 12].

Maintaining normal Kidney function requires a high blood flow and a high glomerular filtration rate (GFR) [13]. Additionally, the proximal tubules need large amount of energy in the process of electrolyte re-absorption [13]. The proximal tubules are thus particularly vulnerable to ischemia and/or hypoxia. Ischemic injury is often an integration of multiple complex pathological processes, including apoptosis and autophagy [13].

Autophagy occurs at basal level in most tissues and contributes to the routine turnover of cytoplasmic components [14, 15]. However, autophagy can also be induced by nutrient deprivation or starvation [14, 15]. In addition to turnover of cellular components, autophagy is also essential for cell homeostasis [16], as well as cell adaptation and defense to adverse environments [17, 18]. Studies have highlighted the importance of autophagy in normal proximal tubule function and recovery from acute ischemic injuries [19–21]. Paradoxically, chronic activation of autophagy may also promote cell damage [19–21].

In the study, e employed the human renal proximal tubular epithelial cell line HK-2, as well as a mouse model of renal ischemia-reperfusion injury to explore the potential function of PHD2 in the process.

## RESULTS

### PHD2 and HIFα expressions are increased in HK-2 tubular epithelial cell after CoCl_2_ treatment

**Figure 1:**
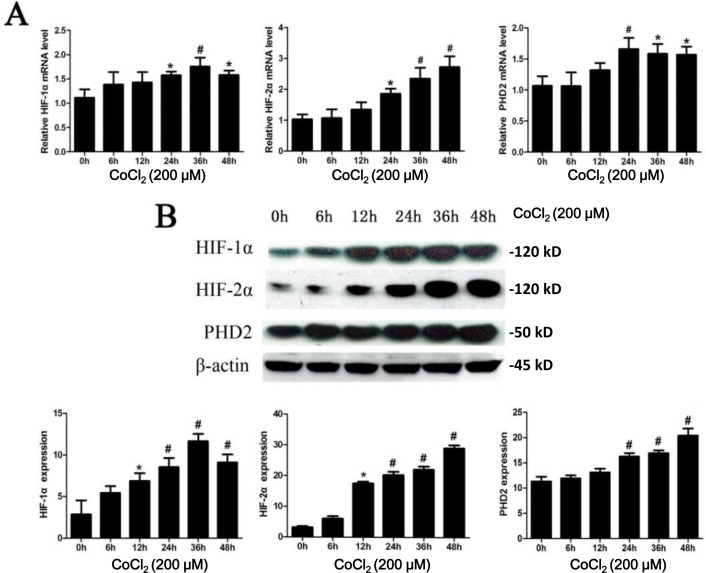
CoCl_**2**_ treatment increases expression of HIF-1/2α and PHD2 **A.** HK-2 cells were incubated with 200 μM of CoCl_2_ for applied time, mRNA and protein expressions of HIF-1α, HIF-2α and PHD2 were tested by RT-qPCR assay (A) and Western blot assay **B.**, respectively. HIF-1α, HIF-2α and PHD2 mRNA expression was normalized to 18S (*n* = 12). HIF-1α, HIF-2α and PHD2 protein expression was normalized to β-actin (B, lower panels, *n* = 14). *P < 0.05 *vs.* 0h group; # *P* < 0.01 *vs.* 0h group.

CoCl_2_ mimics the hypoxic condition and has been validated as a simple tool to examine the molecular mechanisms driven by hypoxia *in vitro* [10]. As shown in Figure 1, protein and mRNA expressions of HIF-1α and HIF-2α were low under normoxic conditions. After CoCl_2_ treatment, there was a time-dependent increase of HIF-1/2α expression at the mRNA level (Figure 1A). A similar pattern of protein expression was observed by Western blot assay (Figure 1B). The protein expression of HIF-1α and HIF-2α both significantly increased, starting from 12h (*P* < 0.05 *vs.* “0h”) (Figure 1B). Whereas HIF-1α and HIF-2α mRNA expressions were induced at later periods (starting from 24h) (Figure 1A). The protein and mRNA levels of HIF-1α reached the peak at 36h (*P* < 0.01 *vs.* “0h”), which then declined (Figure 1A). The protein expression of PHD2 gradually increased after exposure to CoCl_2_, reaching the peak at 48h (Figure 1B). While the PHD2 mRNA level reached its peak at 24h (*P* < 0.01 *vs.* “0h”) (Figure 1A).

### CoCl_2_ induces apoptosis and autophagy activation in HK-2 cells

**Figure 2:**
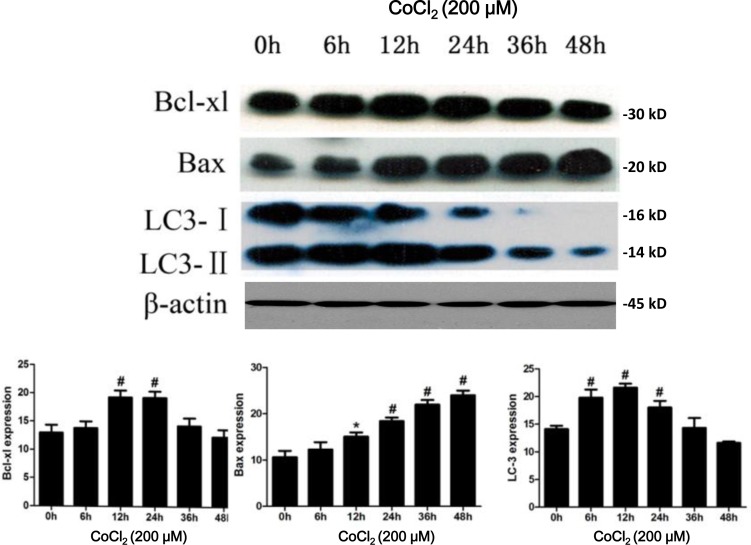
CoCl_**2**_ activates apoptosis and autophagy in HK-2 cells HK-2 cells were incubated with 200 μM of CoCl_2_ for applied time, expressions of listed proteins were tested by Western blot assay (Upper panel), protein expression was normalized to β-actin (low panels, *n* = 12). *P < 0.05 *vs.* 0h group; # *P* < 0.01 *vs.* 0h group.

Next, we tested the expression of Bcl-xL, a known HIF-1α-regulaed gene [22], in CoCl_2_-treated HK-2 cells. As shown in Figure 2, the protein expression of Bcl-xL was increased after CoCl_2_ treatment. It level was peaked at 12-24h and was then declined afterwards (Figure 2). We also analyzed the apoptotic response of HK-2 cells to CoCl_2_. Western blot results in Figure 2 showed that CoCl_2_ up-regulated the pro-apoptotic Bax [23, 24] in HK-2 cells (Figure 2), suggesting apoptosis activation.

**Figure 3:**
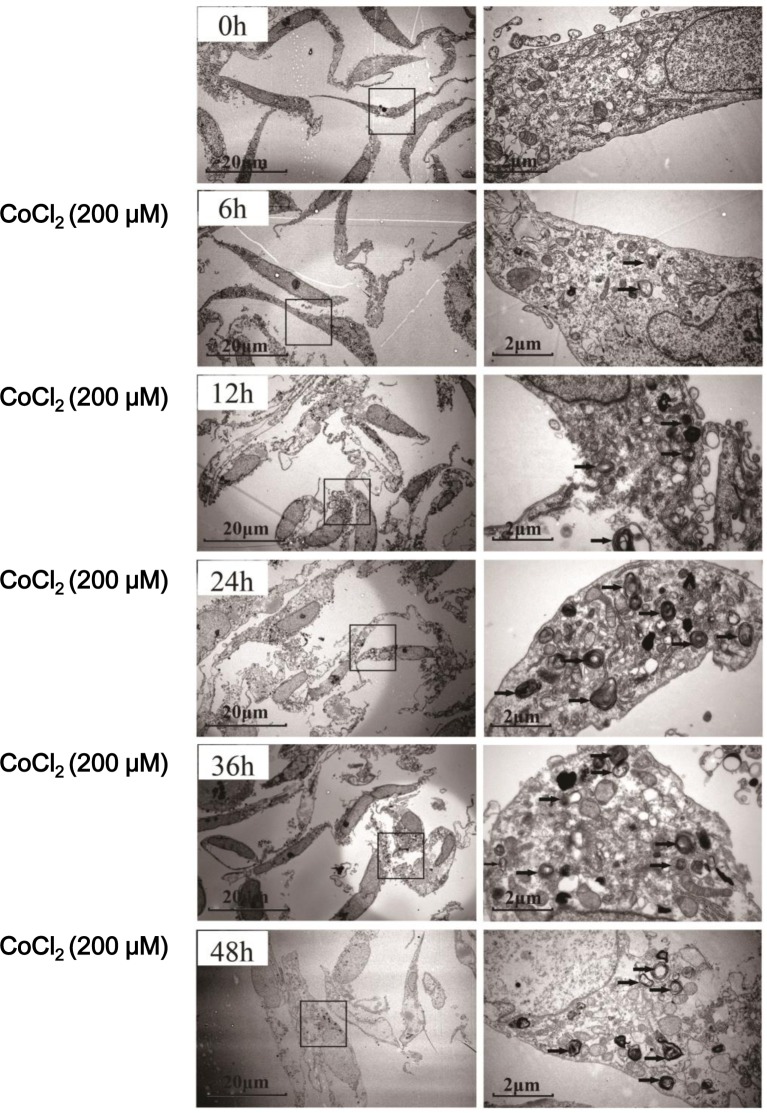
Autophagosome formation in CoCl_**2**_-treated HK-2 cells HK-2 cells were incubated with 200 μM CoCl_2_ for 0-48 h. The cells were then fixed and processed for electron microscopy. Autophagosomes (black arrows) with characteristic double or multiple membranes were identified. Experiments in this figure were repeated four times, and similar results were obtained.

To test whether autophagy could be induced after hypoxia treatment, we investigated the expression of LC3-II, whose upregulation is considered as the characteristic marker of autophagy [25]. CoCl_2_ induced a significant accumulation of LC3 in HK-2 cells. Upregulation of LC3-II was most significant at 12h (*P* < 0.01 *vs.* “0h”) after CoCl_2_ treatment (Figure 2). And its level was then decreased thereafter (Figure 2). We further examined CoCl_2_-induced autophagy in HK-2 cells by transmission electron microscope (TEM) (Figure 3). As shown in the representative micrographs, autophagosomes, with characteristic features of double or multiple membranes, began to appear 6h after CoCl_2_ treatment in the HK-2 cells (Figure 3). They were observed up to 48h after treatment of CoCl_2_ (Figure 3). These results indicate that CoCl_2_ activates autophagy in HK-2 cells.

### Autophagy inhibitor 3-MA inhibits HK-2 cell death and apoptosis by CoCl_2_

**Figure 4:**
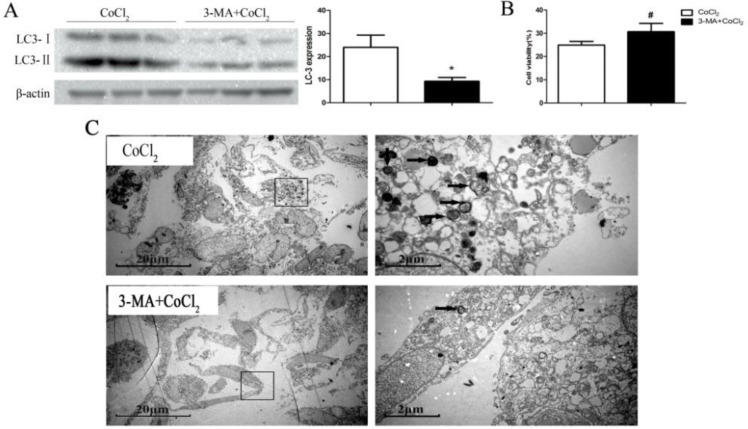
Autophagy inhibitor 3-MA inhibits HK-2 cell death by CoCl_2_ HK-2 cells were pre-incubated with 3-MA (5 mM) for 1h before CoCl_2_ (200 μM) treatment. Samples were collected 24h after treatment, expressions of listed proteins were tested by Western blot assay **A**; Relative cell viability (vs. untreated control cells) was evaluated with Alamar Blue analysis **B.** Electron micrograph images showed the ultrastructure of cells with indicated treatment. *P < 0.01 *vs.* CoCl_2_ only group; # *P* <0.05 *vs.* CoCl_2_ only group.

To explore the role of autophagy in CoCl_2_-induced HK-2 cell death, pharmacological strategy was applied. One hour prior to the CoCl_2_ treatment, we exposed cells to 3-methyladenine (3-MA), which is a selective inhibitor of the autophagy [26]. As shown in Figure 4A and 4B, 3-MA decreased the LC3II/LC3I ratio and increased cell viability compared to the CoCl_2_ only group (24.91±1.59 *vs.* 29.6±2.79, *P* < 0.01). Further, CoCl_2_-induced HK-2 cell apoptosis, tested by ssDNA ELISA assay, was also attenuated by the pretreatment of 3-MA (Supplementary Figure 1A). Additionally, under electron microscopy, the number of autophagosomes decreased and the cell ultrastructure appeared relatively normal (Figure 4C). These results suggested that the autophagic process may be harmful to human renal tubular epithelial cells during CoCl_2_-induced hypoxia.

### PHD2 silencing in HK-2 cells attenuates apoptosis and autophagy by CoCl_2_

**Figure 5:**
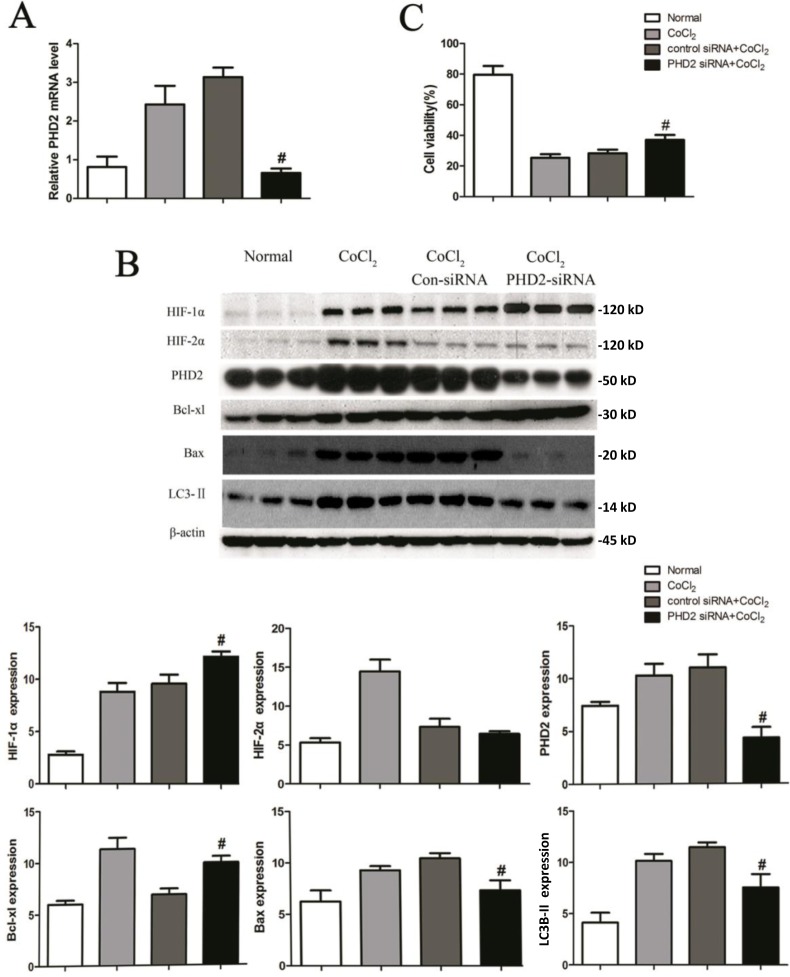
PHD2 silencing in HK-2 cells attenuates apoptosis and autophagy by CoCl_**2**_ HK-2 cells transfected with indicated siRNA were treated with or without CoCl_2_ (200 μM) for 24h, PHD2 mRNA expression was tested by RT-qPCR assay **A**; Expressions of listed proteins were tested by Western blot assay **B**; Relative cell viability (vs. untreated control cells) was evaluated with Alamar Blue analysis **C.** Protein expression was normalized to β-actin (low panels, *n* = 12). ^#^*P* < 0.01 *vs*. Con-siRNA group.

PHD2 is the critical oxygen sensor of HIF-1α [8, 9]. In order to determine the role of PHD2 in autophagy during CoCl_2_ treatment, small interference RNA (siRNA) strategy was applied to knockdown PHD2 in HK-2 cells. Following PHD2 siRNA transfection, mRNA and protein expression of PHD2 decreased by 79% (3.13±0.25 *vs.* 0.66±0.11, *P* < 0.01) and 61% (11.00 ± 1.00 *vs.* 4.32 ± 0.80, *P* < 0.01) respectively (Figure 5A and 5B). HIF-1α protein level was increased by 27% (9.57±0.72 *vs.* 12 ±0.42, *P* < 0.01) (Figure 5B). Yet, there was little change on HIF-2α expression in the same condition (Figure 5B). Intriguingly, PHD2 siRNA up-regulated Bcl-xL, whiling downregulation Bax in HK-2 cells (Figure 5B). Further, Western blot assay revealed that the pattern of LC3II/LC3I expression in the PHD2 siRNA cells was consistent with the 3-MA-treated cells. PHD2 siRNA decreased LC3II expression in CoCl_2_-treated HK-2 cells (Figure 5B). We also studied the influence of silencing PHD2 on cell viability under hypoxia. As shown in Figure 5C, PHD2 siRNA protected HK-2 cells from CoCl_2_ insults (cell viability, 28.32 ±2.41 *vs.* 37.04±3.25, *P* < 0.01). Meanwhile, CoCl_2_-induced HK-2 cell apoptosis was also inhibited by PHD2 siRNA (Supplementary Figure 1B). These results demonstrate that, in CoCl_2_-treated HK-2 cells, PHD2 silencing facilitates HIF-1α expression, and exerts cyto-protective effect possibly via inhibiting apoptosis and autophagy. In line with these findings, we showed that L-mimosine (L-Mim), a non-selective PHD inhibitor [27], also inhibited CoCl_2_-induced viability reduction (Supplementary Figure 1C) and apoptosis activation (Supplementary Figure 1D) in HK-2 cells.

### HIF-1α knockdown abolishes cytoprotection by PHD2 siRNA in CoCl_2_-treated HK-2 cells

**Figure 6:**
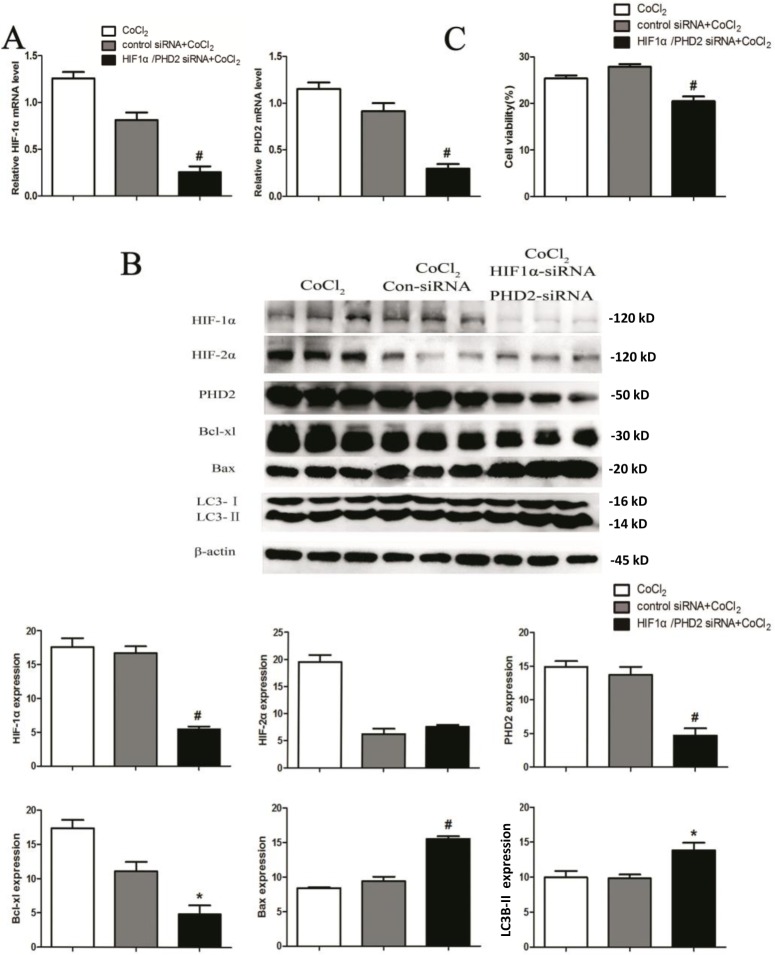
HIF-1α knockdown abolishes cytoprotection by PHD2 siRNA in CoCl_**2**_-treated HK-2 cells HK-2 cells were transfected with applied siRNA (Control siRNA, HIF-1α siRNA or PHD2 siRNA), cells were then treated with CoCl_2_ (200 μM) for 24h, expressions of listed proteins and mRNAs were shown **A.** and **B.** Relative cell viability (vs. untreated control cells) was evaluated with Alamar Blue analysis **C.** Protein expression was normalized to β-actin (low panels, *n* = 12). ^#^ *P* < 0.01 *vs*. Con-siRNA group (A-C).

To further investigate the molecular mechanisms of PHD2/HIF on cell survival under hypoxia, HK-2 cells were co-treated with PHD2 siRNA and HIF-1α siRNA. As shown in Figure 6A, mRNA levels of PHD2 and HIF-1α were decreased by 68% and 69%, respectively (0.91±0.12 *vs.* 0.29 ± 0.09, 0.81±0.22 *vs.* 0.25±0.19; *P* < 0.01) 24h after corresponding siRNA transfection. Protein level of PHD2 and HIF-1α were also decreased by 66% and 67% (*P* < 0.01) (Figure 6B). Simultaneously, silencing PHD2 and HIF-1α also down-regulated Bcl-xL protein (*P* <0.05), yet up-regulating Bax (*P* < 0.01) and LC3II expression (*P* < 0.05). Importantly, HIF-1α siRNA knockdown almost abolished cytoprotection by PHD2 siRNA in CoCl_2_-treated HK-2 cells (Figure 6C).

### L-mimosine administration attenuates renal ischemia-reperfusion injuries

**Figure 7:**
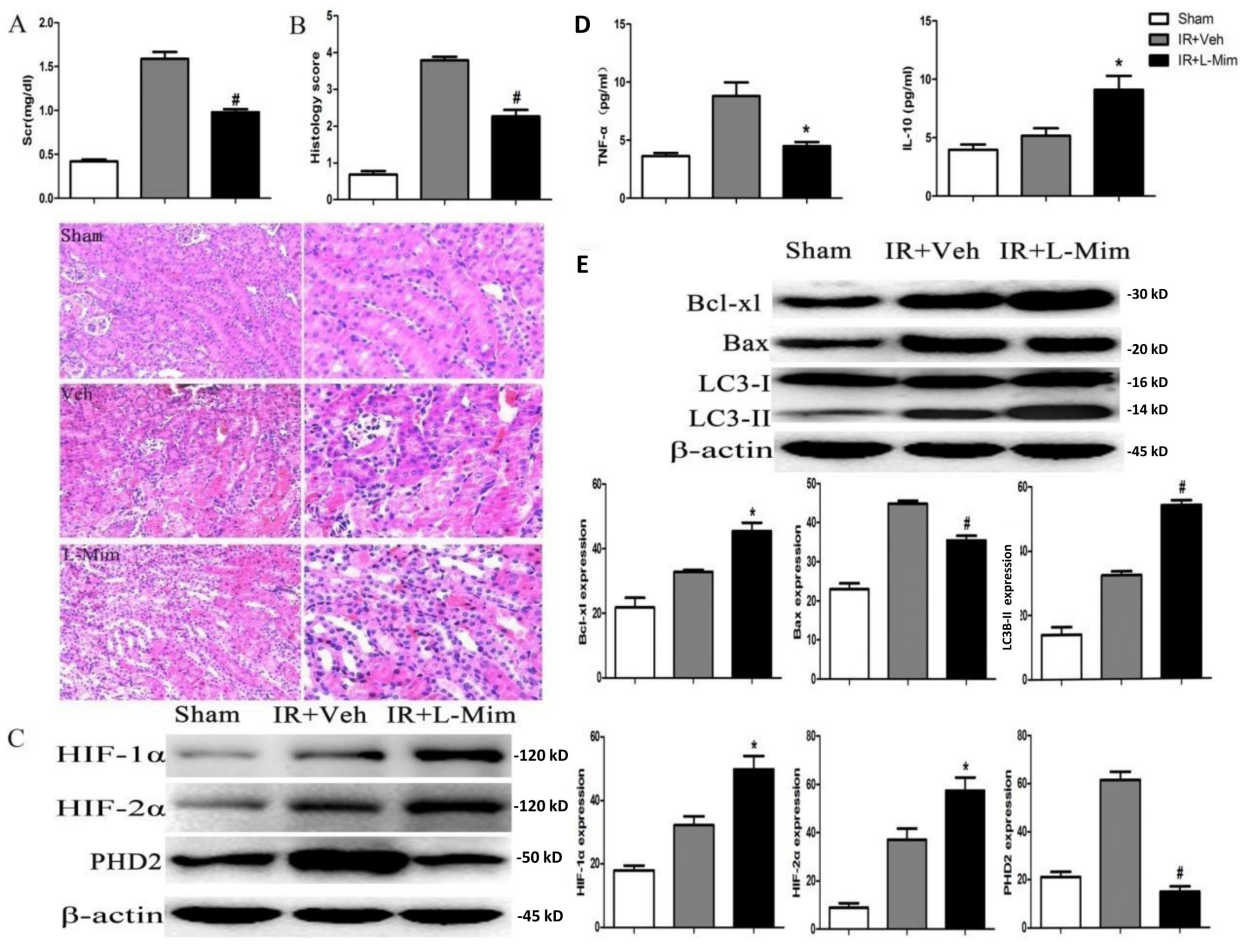
L-mimosine administration attenuates renal ischemia-reperfusion injuries The 6-8 week male C57BL/6J mice, pretreated with L-mimosine (L-Mim, 50 mg/kg, “IR+L-Mim”) or vehicle control (10% NaHCO_3_, “IR+Veh”), were subjected to ischemia reperfusion operation. After 24h, serum creatinine (Scr) level was analyzed **A**; HE staining were performed on kidney slices, and History scores were calculated **B**; Expressions of listed proteins in fresh kidney slices were tested by Western blot **C.** and **E**; Level of TNF-α and IL-10 in the fresh kidney slices was tested by ELISA assay **D.** Sham-operated mice served as controls. Protein expression was normalized to β-actin (*n* = 10). *P < 0.05 *vs*. “IR+Veh” group (C-F); ^#^ *P* < 0.01 *vs*. “IR+Veh” group (C-F);

To study the role of PHD2 *in vivo*, we applied a non-selective PHD inhibitor L-mimosine. Animals in the L-mimosine group (50mg/kg body, 6h before surgery) showed improved renal function compared to those in the vehicle group, and the serum level of creatinine was dramatically decreased in L-mimosine-treated mice (Figure 7A). Histological examinations revealed significant tubular damage and a high injury score in the injured kidney 24h after surgery, which were largely inhibited by L-mimosine pretreatment. (Figure 7B).

For the signaling studies, we showed that L-mimosine administration augmented HIF-1/2α accumulation after ischemia (Figure 7C). HIF-1α and HIF-2α protein expression in kidney increased by 35.4% (32.18±4.75 *vs.* 49.84±7.14, *P* <0.05) and 35.5% (37.02±8.06 *vs*. 57.42±9.44, *P <*0.05) respectively with L-mimosine treatment (Figure 7C). Conversely, the expression of PHD2 was significantly decreased by 76.0% (61.46±5.89 *vs.* 14.78±3.79, *P* < 0.01) after L-mimosine treatment.

ELISA analysis results demonstrated that, with the L-mimosine pre-treatment, the level of inflammatory cytokine TNF-α was markedly decreased, yet the anti-inflammatory cytokine IL-10 was up-regulated (Figure 7D). The above apoptosis and autophagy markers along with inflammatory cytokine levels were also tested *in vivo*. The quantitative analysis revealed that expression of the anti-apoptotic protein Bcl-xL in kidney was up-regulated, but the pro-apoptotic protein Bax was down-regulated following L-mimosine administration (Figure 7E). Further, conversion of LC3-I to LC3-II was up-regulated by L-mimosine treatment, indicating autophagy augmentation (Figure 7E). Collectively, we showed that pretreatment with L-mimosine markedly attenuated the deterioration of renal function and the severity of renal damage. Molecularly, L-mimosine inhibited apoptosis, facilitated autophagy and suppressed inflammatory responses in injured kidney.

## DISCUSSION

The main findings of our study are as follows: PHD2 siRNA knockdown produced a renoprotective effect by attenuating apoptosis and autophagy in HK-2 cells. Further, L-mimosine-activated HIF offered significant protection by attenuating apoptosis, enhancing autophagy and suppressing inflammatory responses in mice.

We showed that treatment of CoCl_2_, a hypoxia mimic, in HK-2 tubular epithelial cells induced PHD2 and HIF-1/2α expression. siRNA knockdown of PHD2 protected HK-2 cells from CoCl_2_ possibly though facilitating HIF-1α expression. Reversely, HIF-1α siRNA knockdown almost abolished cytoprotection by PHD2 siRNA in CoCl_2_-treated HK-2 cells. Therefore, target HIF-1α by modulating PHD2 could offer protection of tubular epithelial cells in hypoxia conditions.

Autophagy could be cell detrimental or cytoprotective depending on the stimuli that provoked it. It is still largely unknown what mechanisms determine the final outcomes [14–16]. Here, we observed that CoCl_2_ activated autophagy in HK-2 cells. On the other hand, 3-MA, the autophagy inhibitor, blocked autophagy and protected HK-2 cells from CoCl_2_. These results indicate that the autophagic pathway may act as a detrimental factor in the CoCl_2_ hypoxic model at least *in vitro*, although more detailed studies are still needed.

Interestingly, our *in vivo* results demonstrated that autophagy induction was accompanied with improved kidney functions. These results indicated that autophagy may play a protective role against kidney ischemia reperfusion injuries. These *in vivo* results are consistent with other studies [28, 29]. The mechanisms underlying the pro-survival effect of autophagy remain largely unknown [28, 30]. In the process of renal ischemia reperfusion injury, autophagy activation was shown to eliminate damaged mitochondria and prevent accumulation of aggregate-prone proteins [28, 29]. The rapid increased number of research publications on autophagy and hypoxia will lead to a better understanding the mechanisms involved.

Collectively, the results of our study suggest that activating HIF, i.e. via inhibiting PHD, may be a useful therapeutic strategy in ischemic renal injury.

## MATERIALS AND METHODS

### Cell culture and treatment

HK-2, a human kidney epithelial cell line [31], was purchased from American Type Culture Collection (Manassas, VA). Cells were cultured in DMEM/F12 (Gibco, Invitrogen, USA) with 10% fetal bovine serum (FBS, Gibco, Invitrogen, USA) under 5% CO_2_ and 95% air atmosphere at 37°C. Cells were plated onto 35-mm dishes at a density of ~0.5 ×10^6^ cells/dish until 70-80% confluent for experiment.

### PHD2 and HIF-1α gene silencing

Small interfering RNA (siRNA) sequences against HIF-1α, PHD2 and a scrambled control siRNA were designed and synthesized by GenePharma (GenePharma, Shanghai, China). siRNAs were transfected using DharmaFECT 1 (Dharmacon, Shanghai, China) as previously described [32]. Transfected cells were incubated at 37°C for 24h. Protein and RNA expressions were analyzed Western blot and RT-qPCR respectively.

### Protein extraction

Cells were grown to 70-80% confluence and stimulated with 200 μM CoCl_2_ for the indicated time period. Afterwards, cells were placed on ice immediately and washed with PBS, and cell lysis was performed using 8 M urea, 10% glycerol, 10 mM Tris-HCl (pH 6.8), 1% SDS, 5mM dithiothreitol, and the Complete Mini Protease-Inhibitor Cocktail (Roche, Mannheim, Germany). After being centrifuged at 12,000g for 15 min at 4°C, the supernatant was collected, and protein concentrations were determined. The protein concentration was determined by using a Bio-Rad Bradford protein assay kit (Bio-Rad, Shanghai, China). HIF-1α antibody (3716), HIF-2α antibody (7096), PHD antibody (3293), Bax antibody (2772), Bcl-xL antibody (2762), LC3B antibody (2775) and β-actin antibody (3700) were purchased from Cell Signaling Tech (Shanghai, China). Band intensity (total gray) was quantified via the ImageJ software (NIH), the values were always normalized to corresponding loading control.

### Assay of cell viability

For cell viability assessment, 10 μL of Alamar Blue (Kaiji, Nanjing, China) was added to each well and the plates were incubated at 37°C and 5% CO_2_ for 2h. The absorbance was measured by using a Synergy HT multi-detection microplate reader (Bio-Tek, Winooski, VT) at a test wavelength of 570 nm.

### Apoptosis assay by single strand DNA (ssDNA) ELISA

DNA denature is an important characteristic marker of cell apoptosis. Following the treatment of cells, denatured ssDNA was detected through a nucleosomal monoclonal antibody in an ELISA format. Briefly, cells (1×10^4^/well) were seeded onto 96-well plates. Cell apoptosis was analyzed by the ssDNA ELISA kit from Chemicon International (Temecula, CA, Catalog Number: 1217). OD value at 450 nm was utilized as a quantitative indicator of cell apoptosis.

### Electron microscopy

Cells were grown in T25 flasks and fixed for 4h in 2.5% glutaraldehyde fixative in phosphate buffer at 4°C. After fixation, cell monolayers were placed in glutaraldehyde wash solution and then post-fixed in Millonigs Osmium Tetroxide fixative for 15-30 minutes. The cultures were dehydrated through a graded series of 10% to 100% ethanol, infiltrated, and then embedded in TAAB Premix Resin medium. Ultrathin sections were collected on copper grids and stained with uranyl acetate and lead citrate. Sections were viewed using a Hitachi H7600 transmission electron microscope (TEM).

### Real-time reverse transcription-polymerase chain reaction (RT-qPCR)

Total RNA of HK-2 cells was extracted using Trizol reagents (Invitrogen, Carlsbad, CA). Extracted RNA was reverse-transcribed to complementary DNA (PrimeScript; TaKaRa, Kyoto, Japan), which was utilized for real-time polymerase chain reaction (PCR) (SYBR reagents, TaqTMTaKaRa). PCR primers (Sangon, Shanghai, China) were designed with the following sequences: HIF-1α forward: 5′-CGGAGGTGTTCTATGAGCTGG-3′, reverse: 5′-AGCTTGTGTGTTCGCAGGAA-3′. HIF-2α forward: 5′-CGGAGGTGTTCTATGAGCTGG-3′, reverse: 5′-AGCTTGTGTGTTCGCAGGAA-3′. PHD2 forward: 5′-AGGCGATAAGATCACCTGGAT-3′, reverse: 5′-TTCGTCCGGCCATTGATTTTG-3′; 18s forward: 5′-CGGCTACCACATCCAGAA-3′, reverse: 5′-CCTGTATTGTTATTTTTCGTCACTACCT-3′. 18s mRNA was tested as endogenous control for desired mRNAs. The relative gene expressions were calculated in accordance with the ^ΔΔ^Ct method [33]. Relative mRNA levels were expressed as 2^−ΔΔ^Ct and ratios to internal control.

### Mouse renal ischemia reperfusion injury

Experiments were performed on 6-8 week male C57BL/6J mice weighing 20-22 g (from Animal Center of Fudan University, Shanghai, China). Animals were housed in temperature- and humidity-controlled cages, with free access to water and rodent food on a 12-h light/dark cycle. All protocols were approved by the Institutional Animal Care and Use Committee of Fudan University, and adhered strictly to the NIH Guide for the Care and Use of Laboratory Animals. All surgery was performed under sodium pentobarbital anesthesia, and efforts were made to minimize suffering. L-mimosine (L-Mim, Sigma, Dorset, UK) was resolved in 10% NaHCO_3_ at a concentration of 15 mg/mL (adjusted pH to 7.4 by HCl plus PBS) and administered intraperitoneally at 50 mg/kg, 6h before renal ischemia reperfusion injury surgery.

Mice were anesthetized with intraperitoneal sodium pentobarbital (30mg/kg), and renal ischemia reperfusion injury was induced by bilateral renal pedicle clamping for 35 min. Sham-operated mice underwent the same surgical procedures but with no occlusion of the renal pedicle. Intra-rectal temperature of mice was maintained at 36.5°-37.0°C with a heating pad. Blood and kidney samples of all mouse groups were harvested at 24h after the surgery. Animals were divided into three groups (*n* = 4): Group 1 (Sham): Sham-operated animals without induction of ischemia reperfusion injury; Group 2 (Veh): Animals pretreated with NaHCO_3_ (PH 7.4) 6h before of ischemia reperfusion injury; Group 3 L-mimosine (L-Mim, Sigma, M0253, Shanghai, China) treatment group: Animals pretreated with L-Mim 6h before of ischemia reperfusion injury;

### Measurement of renal functions and histological analysis

Blood samples were obtained at time of sacrifice for measurement of serum creatinine (Scr). Scr level was determined using the Quantichrom Creatinine Assay Kit (DICT-500, Universal Biologicals, Shanghai, China), following the manufacturer's protocol. Kidney slices were embedded in paraffin wax, sectioned at 4 μm and stained with hematoxylin and eosin (HE) with standard methods. Histopathological scoring was performed in a blinded manner by a pathologist without knowing the experimental protocol. Severity of tubular injury was scored on a semi-quantitative scale: “0” = normal, “1” = 10-25%, “2” = 26-50%, “3” = 51-75%, and “4” = >75%. At least 10 consecutive fields in the corticomedullary junction and outer medulla were examined under × 400 magnification (Lecia, DM6000 B, Germany).

### Enzyme-linked immunosorbent assay (ELISA)

The levels of pro-inflammatory factor tumor necrosis factor-α (TNF-α) and anti-inflammatory cytokine Interleukin-10 (IL-10) were measured by ELISA (MBS495354 for TNF-α, and MBS288580 for IL-10, BioSource, Shanghai, China). Photometric measurements were conducted at 450 nm via a microplate reader (Bio-Rad).

### Western blot assay

Protein lysates (30 μg protein per sample) were loaded and separated on sodium dodecyl sulfate-polyacrylamide gels (SDS-PAGE) and then transferred to a polyvinylidene difluoride (PVDF) membrane. The membrane was blocked with 5% nonfat milk and incubated with primary antibodies and corresponding second antibodies. Following primary antibodies were utilized in this study: HIF-1α (mouse monoclonal 1:500; Abcam, Cambridge, UK), HIF-2α (rabbit monoclonal 1:500; Novus, Littleton, CO), PHD2 (rabbit monoclonal 1:1500; Novus, Littleton, CO), Bcl-xL (rabbit monoclonal 1:1,000; Cell Signaling Technology, Danvers, MA), Bax (rabbit monoclonal 1:1,000; Cell Signaling Technology, Danvers, MA) or Light chain 3 (LC-3, rabbit monoclonal 1:1,000; Cell Signaling Technology, Danvers, MA).

### Statistical analysis

Statistical analysis was performed using the statistical software SPSS Version 20.0 (SPSS Inc., Chicago, IL). Data were analyzed using the *t* test when comparing two groups and one-way ANOVA followed by Tukey *t*-test for the time course data. *P* <0.05 was considered significant. Data were shown as mean ± SEM.

## SUPPLEMENTARY MATERIALS


