# Supplementary material for: Prolyl hydroxylase 2 (PHD2) inhibition protects human renal epithelial cells and mice kidney from hypoxia injury
Source: Oncotarget. 2016 Aug 5;7(34):54317–28. doi: 10.18632/oncotarget.11104 (PMC5342344; doi:10.18632/oncotarget.11104)
Supplement: Supplementary file 1 [file oncotarget-07-54317-s001.pdf]

Prolyl hydroxylase 2 (PHD2) inhibition protects human renal epithelial cells and mice kidney from hypoxia injury

Supplementary Material

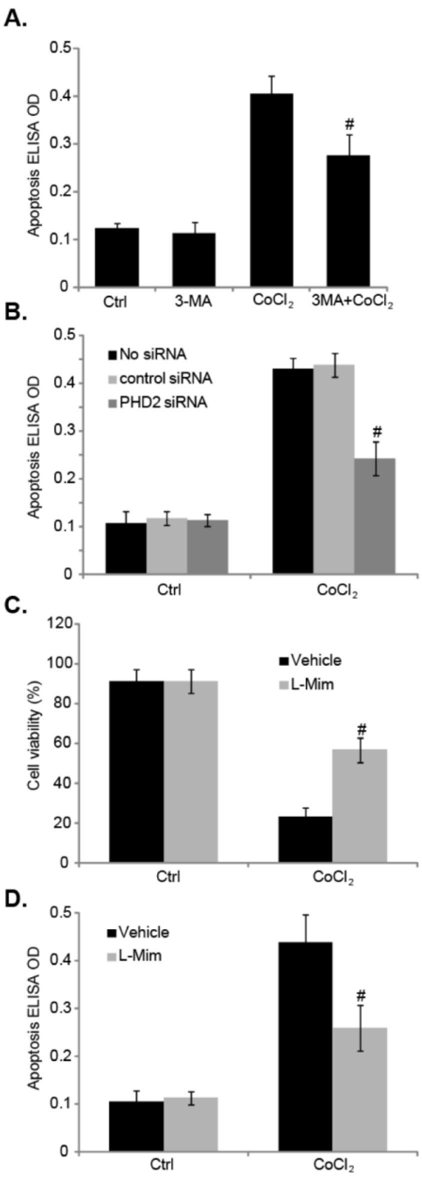

Supplementary Figure 1.

HK-2 cells were pre-incubated with 3-MA (5 mM) for 1h before $CoCl_2$ (200 μM) treatment for 24h, cell apoptosis was tested by ssDNA ELISA assay (**A**). HK-2 cells transfected with indicated siRNA were treated with or without

$CoCl_2$ (200 μM) for 24h, cell apoptosis was tested by ssDNA ELISA assay (**B**). HK-2 cells were pre-incubated with 200 μM of L-mimosine (L-Mim), followed by $CoCl_2$ (200 μM) treatment for 24h, cell viability was tested by the Alamar Blue assay (**C**), and cell apoptosis was tested by ssDNA ELISA assay (**D**). "Vehicle" stands for 0.1% of DMSO (**C** and **D**). $^{\#}$ ***P*** <0.01 vs. $CoCl_2$ only group (**A**, **C** and **D**). $^{\#}$ ***P*** <0.01 vs. control siRNA group (**B**).
